# Differential impact of bisphenol S on human arterial endothelial cells from healthy and type-2 diabetic donors: a high-content imaging study

**DOI:** 10.1007/s00418-026-02535-0

**Published:** 2026-09-09

**Authors:** Andrea di Credico, Giulia Gaggi, Sandra Bibbò, Marco Marchisio, Angela di Baldassarre, Barbara Ghinassi

**Affiliations:** 1 Department of Medicine and Aging Sciences, G. d’Annunzio University of Chieti-Pescara, Via Dei Vestini 31, 66100 Chieti, Italy; 2 Cell Reprogramming and Differentiation Laboratory, Center of Advanced Studies and Technologies (CAST), G. d’Annunzio University of Chieti-Pescara, 66100 Chieti, Italy; 3 UdA-Tech Lab, G. d’Annunzio University of Chieti-Pescara, 66100 Chieti, Italy; 4 Department of Innovative Technologies in Medicine and Dentistry, G. d’Annunzio University of Chieti-Pescara, 66100 Chieti, Italy

**Keywords:** Endothelial dysfunction, Type 2 diabetes, Bisphenol S, High-content imaging, Phenotype classification, Cell Painting

## Abstract

Endothelial dysfunction is a central mechanism linking type 2 diabetes (T2D) to cardiovascular complications, and environmental endocrine-disrupting chemicals may further perturb vascular homeostasis. Bisphenol S (BPS), widely used as a bisphenol A substitute, is increasingly detected in human and environmental samples, but its impact on endothelial morphology in the context of pre-existing metabolic disease remains poorly defined. Here, we used high-content imaging and Cell Painting to determine whether exposure to 0.1 µM BPS for 96 h induces distinct morphology-based responses in primary human arterial endothelial cells from healthy and T2D donors. Multiplex staining of DNA, RNA-rich regions, RER, Golgi/membrane structures, and mitochondria was combined with automated segmentation, multidimensional feature extraction, sparse partial least-squares discriminant analysis, and inferential statistics. Metabolic status was the main determinant of the endothelial morphology-based profile, clearly separating healthy and T2D-derived cells. In addition, BPS induced a pronounced multidimensional phenotypic shift in healthy endothelial cells, involving nuclear, mitochondrial, RNA-associated, and membrane/perinuclear descriptors. In contrast, T2D-derived cells retained a dominant disease-associated profile, with a less evident whole-cell response to BPS. However, selected RER-associated descriptors remained BPS-responsive, indicating that BPS can still modulate stress-related subcellular organization under diabetic conditions. These findings show that BPS perturbs endothelial morphology under exposure-relevant low-dose conditions but that baseline metabolic status strongly shapes the magnitude and compartmental distribution of this response. The study identifies the diabetic endothelial phenotype as a key modifier of BPS-induced cellular remodeling and supports Cell Painting as a sensitive strategy to resolve context-dependent endothelial responses to environmental pollutants.

**Supplementary Information:**

The online version contains supplementary material available at https://doi.org/10.1007/s00418-026-02535-0.

## Introduction

Cardiovascular disease is the leading cause of morbidity and mortality in individuals with type 2 diabetes (T2D), and endothelial dysfunction is a central mechanism linking metabolic dysregulation to vascular complications (Wong and Sattar 2023). In diabetic conditions, endothelial cells progressively lose their ability to maintain vascular homeostasis, with impaired nitric oxide bioavailability, increased oxidative stress, mitochondrial dysfunction, inflammatory activation, altered permeability, and pro-thrombotic remodeling (Magenta et al. 2014; Xue et al. 2023; Yang et al. 2024). Because the diabetic endothelium is already exposed to chronic metabolic stress, identifying additional environmental factors that may further compromise the endothelial phenotype is highly relevant for understanding cardiovascular vulnerability in T2D.

Environmental endocrine-disrupting chemicals (EDCs) are increasingly recognized as potential contributors to cardiometabolic disease. Among them, bisphenol S (BPS) has been widely introduced as a substitute for bisphenol A (BPA), following growing toxicological and regulatory concern regarding BPA exposure (Rochester and Bolden 2015). In the European Union, BPA use has been banned or restricted in several food-contact materials based on updated risk assessment, but replacement bisphenols, including BPS, remain less extensively characterized. This raises concern that BPA substitution may have introduced alternative compounds with similar biological activity but incomplete toxicological evaluation. Consistently, BPS shares structural similarities with BPA and has been reported to interfere with endocrine signaling, oxidative balance, mitochondrial function, and metabolic regulation (Wu et al. 2018; Catenza et al. 2021).

Human exposure to BPS is now well documented. BPS has been detected in urine samples from different countries, supporting widespread population exposure, and more recent evidence has reported its presence in serum and whole blood, with detection frequencies of 49% and 78%, respectively, and concentrations up to 1.7 and 2.1 ng/ml (Liao et al. 2012; Fu et al. 2024). These observations support the use of low-dose experimental models to approximate biologically plausible exposure conditions. Previous work from our group has shown that BPS and related plastic-associated pollutants can exert measurable biological effects in human stem cell-based models at environmentally relevant concentrations. In human-induced pluripotent stem cells and perinatal stem cells, exposure to bisphenols and perfluoroalkyl substances was associated with altered proliferation, mitochondrial impairment, and changes in genes involved in pluripotency, germline specification, and epigenetic regulation (Gaggi et al. 2023, 2024; Di Credico et al. 2023a). These findings support the concept that BPS can perturb fundamental cellular programs beyond classical endocrine endpoints and provide a rationale for investigating whether similar low-dose exposure can also affect vascular cells.

Although most vascular studies have focused on BPA, emerging evidence indicates that BPS can also affect endothelial biology. In human endothelial cells and experimental models, BPS exposure has been linked to oxidative stress, altered nitric oxide signaling, inflammatory activation, and mitochondrial dysfunction (Easson et al. 2022). These mechanisms overlap with pathways already dysregulated in diabetes, suggesting that BPS exposure may not act as an isolated toxic stimulus but rather as a potential additional stressor in a pre-existing disease-associated endothelial phenotype.

This issue is particularly relevant because endothelial cells derived from diabetic donors can retain persistent structural and functional alterations, including changes in cytoskeletal organization, intracellular trafficking, mitochondrial function, and stress-response pathways (Gorashi et al. 2023). Such baseline differences may influence how cells respond to environmental perturbations. Therefore, a key unanswered question is whether BPS induces comparable endothelial remodeling in healthy and diabetic cells or whether the pre-existing diabetic phenotype constrains, masks, or redirects the cellular response to this pollutant.

Detecting these effects requires tools capable of capturing subtle, multidimensional changes in cellular organization. Traditional assays based on single endpoints may overlook coordinated morphological remodeling induced by environmental exposures. High-content imaging (HCI), and in particular Cell Painting, provides a broad phenotypic profiling strategy that quantifies multiple cellular compartments, including nuclei, mitochondria, endoplasmic reticulum, RNA-rich regions, and membrane systems (Mattiazzi Usaj et al. 2016; Bray et al. 2016; Yin et al. 2020). This strategy is particularly relevant to EDC research, as previous HCI- and machine-learning-based studies from our group have shown that BPS and related pollutants can induce subtle yet classifiable phenotypic alterations in human neuronal cells, even in the absence of overt cytotoxicity (Vulliard et al. 2022; Di Credico et al. 2023b). By combining multiplex imaging with multivariate analysis, this approach can reveal global and compartment-specific phenotypic signatures that may not be evident using conventional assays.

In this study, we applied high-content imaging and Cell Painting to investigate the effect of 0.1 µM BPS exposure (a concentration selected to model exposure-relevant conditions) on primary human arterial endothelial cells from healthy donors and donors with T2D. We aimed to determine whether BPS modifies morphology-based phenotypic features of endothelial cells at the level of the overall cellular profile or specific subcellular compartments and whether this response differs according to baseline metabolic status. By comparing healthy and T2D-derived endothelial cells, we tested the hypothesis that pre-existing diabetic endothelial remodeling modulates the cellular response to BPS and may reveal context-dependent pollutant effects relevant to cardiovascular risk.

## Materials and methods

### Cell culture and maintenance

Primary human arterial endothelial cells (aortic) from healthy donors (LOT 23TL072011, 22TL073027, 25TL282592) and donors with type 2 diabetes (T2D) (LOT 0000239311, 0000239340, 0000253459) were obtained from Lonza Biosciences (Walkersville, MD, USA) and cultured according to the supplier’s recommendations. Three lots were used for each condition. The classification as healthy- or T2D-derived was based on the donor information supplied by the manufacturer. Cells were maintained in Endothelial Basal Medium-2 (EBM-2; Lonza) supplemented with the EGM-2 BulletKit (Lonza), yielding a complete medium containing 2% fetal bovine serum, recombinant human VEGF, EGF, FGF-basic, IGF-1, heparin, hydrocortisone, l-glutamine, and ascorbic acid.

Cultures were incubated at 37 °C in a humidified atmosphere containing 5% CO₂ and subcultured at approximately 80% confluence using Trypsin-EDTA solution (0.05% trypsin, 0.02% EDTA; Corning, New York, NY, USA). Cells were fed every 48 h with fresh complete medium.

### Cell painting assay

After 96 h exposure to 0.1 µM BPS or vehicle [methanol 0.129%, a concentration that has been shown to be non-toxic in previous studies (Nguyen et al. 2020; Di Credico et al. 2023b)], human vascular endothelial cells from healthy donors and with T2D were processed using a multiplexed cell painting-based staining protocol for HCI. The BPS concentration used in this study was selected to model exposure-relevant low-dose conditions (Liao et al. 2012). All experiments were performed using cells between passages 4–5, at around 60% confluency.

The assay was designed to simultaneously visualize major intracellular compartments relevant to endothelial phenotype, including nuclear DNA, RNA-rich regions, rough endoplasmic reticulum, Golgi/membrane system, and mitochondria. The staining was performed as previously described (Gaggi et al. 2025). Briefly, 3 × 10^3^ cells per well were seeded in technical triplicate in PhenoPlate™ 96-well microplates. Three independent donor-derived cell preparations were included for each group (healthy vs T2D). Features obtained from technical replicate wells were averaged, yielding one value per donor and experimental condition. The donor was therefore considered the biological experimental unit, with *n* = 3 donors per condition.

After 96 h treatment with BPS or vehicle control, cells were stained by PhenoVue Cell Painting kit (Revvity, Waltham, MA, USA) according to the manufacturer’s instructions. Mitochondrial dye was first added to the cells, which were then incubated for 30 min at 37 °C, followed by fixation with 3.2% paraformaldehyde. After washing, a mix containing concanavalin A (endoplasmic reticulum), nucleic acid stain (RNA), wheat germ agglutinin (WGA) stain (Golgi apparatus), and Hoechst 33,342 (nuclei) was added to each well and incubated for 30 min. Images were acquired by an Operetta CLS High-Content Analysis System and analyzed using Harmony software (Revvity).

### Data acquisition and high-content imaging workflow

Fluorescence images were acquired using the Operetta CLS High-Content Imaging System (Revvity) at 40 × or 20 × magnification in multiple fluorescence channels to capture the Cell Painting panel. Images were processed using Harmony software for automated segmentation and quantitative feature extraction.

The analytical workflow included image acquisition, automated identification of nuclei and cell bodies, generation of subcellular compartments for region-specific analysis, feature extraction, and data cleaning. Nuclei were identified using the Hoechst signal as the primary segmentation object. Nuclear masks were used to define the position, size, and morphology of individual nuclei and to provide reference objects for subsequent cell-level segmentation.

Starting from nuclear masks, whole-cell and cytoplasmic regions were delineated using cytoplasmic Cell Painting signals. Additional membrane-associated and perinuclear/ring regions were generated to capture subcellular spatial organization. For each identified cell, Harmony extracted morphology-related features from all fluorescence channels and compartments, including signal intensity, texture, symmetry, threshold-based area coverage, and shape descriptors. Features were computed separately for nuclear, cytoplasmic, membrane-associated, and perinuclear/ring regions.

To ensure biologically coherent feature assignment, Hoechst-derived features were retained only for nuclear compartments, whereas the features derived from the other Cell Painting channels were considered in compartments consistent with their biological localization. Extracted features were subsequently used for multivariate analysis, hierarchical clustering, and inferential statistics to identify morphology-based signatures associated with metabolic status and BPS treatment.

### Statistical analysis

High-content imaging data were median-normalized before further analysis. Data normalization, sparse partial least-squares discriminant analysis (sPLS-DA), dendrogram generation, and hierarchical clustering were performed using R scripts implemented in MetaboAnalyst 6.0.

Multivariate analyses were performed using MetaboAnalyst 6.0 with the default software settings.

For feature-level inference, a separate two-way ANOVA model was fitted for each morphological feature, with cell type (healthy vs T2D-derived) and treatment (CTRL vs BPS) as fixed factors. When a significant interaction or main effect was detected, Tukey-adjusted pairwise comparisons were performed.

Statistical analyses were performed using GraphPad Prism. Results were considered statistically significant at *p* < 0.05.

## Results

### Experimental design and workflow

**Fig. 1:**
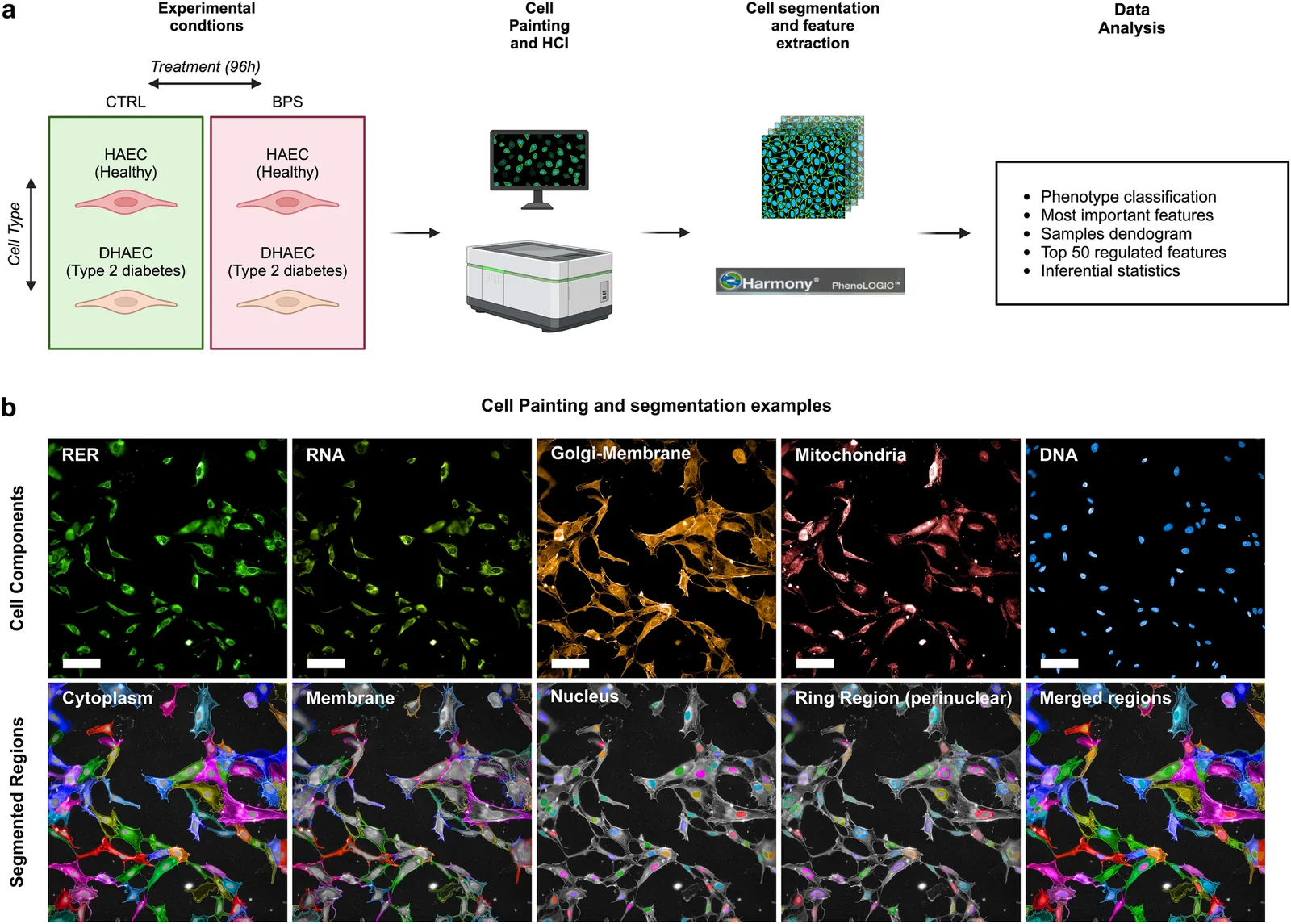
**a** Experimental design and high-content imaging workflow. Healthy and T2D-derived endothelial cells were treated for 96 h with 0.1 µM BPS or vehicle control, stained using the Cell Painting assay, and imaged with the Operetta CLS high-content imaging system. Automated segmentation and feature extraction were performed, followed by multivariate and inferential analyses to generate morphology-based phenotypic profiles. Panel A was created with *BioRender.com*. **b** Representative images of the cellular compartments stained by Cell Painting (upper row) and corresponding segmentation masks generated using Harmony software (lower row). Scale bar, 100 µm; 20 × magnification

Human arterial endothelial cells from healthy donors and from individuals with type 2 diabetes were exposed for 96 h to 0.1 µM BPS or vehicle control, generating four experimental conditions defined by metabolic status and treatment (Fig. 1a). Following exposure, cells were stained using the Cell Painting assay and imaged with Operetta CLS high-content microscopy. Acquired images were subjected to automated cell segmentation and feature extraction, enabling the quantification of morphology-related descriptors across multiple cellular compartments. These features were subsequently used for phenotypic classification, identification of the informative morphological parameters, hierarchical clustering, and statistical inference to characterize treatment- and disease-associated phenotypic patterns. Figure 1 illustrates the experimental design and analytical pipeline adopted to generate high-dimensional morphological profiles.

The fluorescence imaging strategy captured a broad range of cellular compartments, including RER, RNA-rich regions, Golgi membrane structures, mitochondria, and nuclear DNA (Fig. 1b, upper row).

Automated image analysis segmented individual cells and partitioned them into nuclear, cytoplasmic, membrane-associated, and perinuclear/ring regions (Fig. 1b, lower row), allowing region-specific quantification of intensity, texture, spatial distribution, and shape-related features. A merged visualization of all Cell Painting channels, including magnified insets illustrating the overlap among fluorescence signals and the spatial organization of the labeled compartments, is shown in Supplementary Fig. 1. The merged image illustrates the spatial relationship among the Cell Painting signals. Because the RER, Golgi apparatus, and RNA-rich structures are concentrated in closely apposed perinuclear regions, overlap in the merged image does not indicate fluorescence bleed-through or molecular colocalization. Quantitative features were extracted separately from the individual fluorescence channels.

### BPS induces a distinct morphology-based phenotypic shift in healthy endothelial cells, whereas T2D-derived cells retain a dominant disease-associated profile

**Fig. 2:**
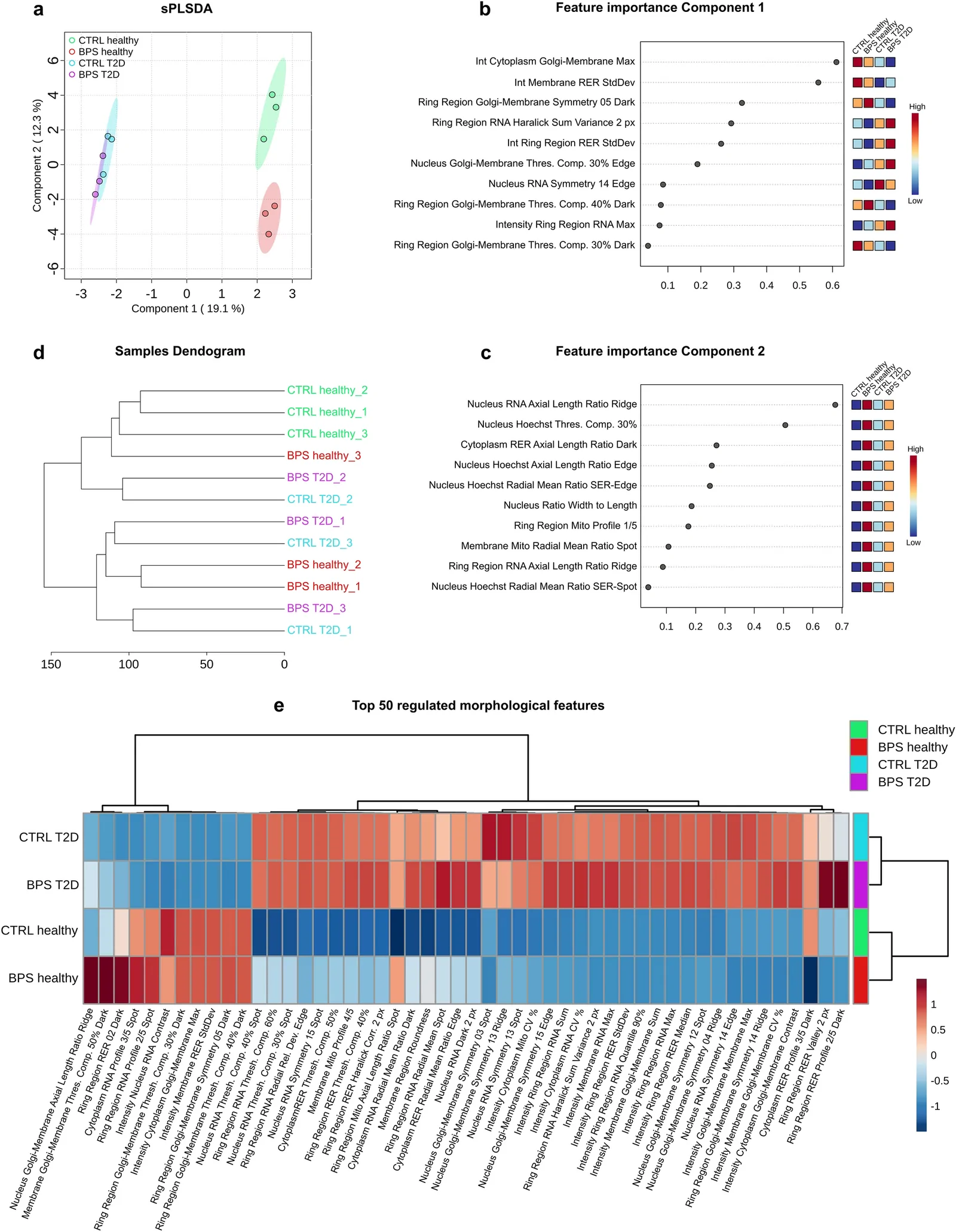
Multivariate morphological analysis of CTRL and BPS-treated cells under healthy and T2D conditions. **a** Sparse partial least squares discriminant analysis (sPLS-DA) performed on the complete set of morphological features shows separation of samples according to metabolic condition and treatment. The first two components explain 19.1% and 12.3% of the total variance, respectively. **b**–**c** Feature importance plots for Components 1 and 2 highlighting the main variables contributing to sample discrimination. **d** Hierarchical clustering of samples based on selected morphological features. **e** Heatmap representation of the top 50 regulated morphological parameters with unsupervised clustering across experimental groups. Data represent three independent biological replicates per condition

To assess whether BPS exposure alters the global cellular phenotype under healthy and diabetic conditions, a sparse partial least squares discriminant analysis (sPLS-DA) was performed on the full set of morphological features. This multivariate approach revealed a clear separation between healthy and T2D cells, indicating that metabolic status is the primary determinant of the cellular morphological profile (Fig. 2a).

Along Component 1, which accounted for 19.1% of the total variance, healthy and T2D-derived samples segregated into two distinct clusters, highlighting marked differences in their overall morphology-based profiles. Within the healthy group, BPS treatment induced a clear phenotypic shift, with BPS-treated healthy cells separating from untreated healthy controls. In contrast, BPS-treated T2D-derived cells clustered in close proximity to untreated T2D-derived cells, suggesting a more limited effect of BPS on the overall multivariate profile under diabetic conditions. Component 2, explaining an additional 12.3% of the variance, further contributed to sample separation and captured additional intra-group variability. Together, the first two components accounted for 31.4% of the total variance and indicated that BPS exposure was associated with a more pronounced morphology-based shift in healthy endothelial cells than in T2D-derived cells (Fig. 2a).

To identify the morphological features contributing to the separation observed in the sPLS-DA, we examined the variables with the highest loadings on the first two components. Component 1, which mainly discriminated healthy from T2D-derived cells and captured the BPS-associated shift in healthy cells, was driven predominantly by features related to intracellular membrane organization and RNA distribution. In particular, intensity- and texture-based descriptors of Golgi membrane structures and RER, together with perinuclear RNA intensity and symmetry parameters, contributed substantially to this component (Fig. 2b). These findings suggest that differences in membrane-associated compartments and RNA spatial organization are major contributors to the multivariate separation among experimental groups.

Component 2 captured additional variability related mainly to nuclear morphology and Hoechst-derived signal distribution. High-loading variables included nuclear axial length ratios, width-to-length ratios, and Hoechst intensity-distribution features, consistent with differences in nuclear shape and chromatin-associated staining patterns (Fig. 2c). These parameters further contributed to the distinction between healthy and T2D-derived cells and to the characterization of treatment-associated morphological variation.

Consistent with the sPLS-DA, hierarchical clustering of samples based on selected morphological features showed segregation according to metabolic status. Healthy control samples clustered together, whereas BPS-treated healthy cells formed a distinct subcluster, supporting the presence of a BPS-associated phenotypic shift in healthy endothelial cells. Conversely, BPS-treated T2D-derived samples clustered close to untreated T2D-derived cells, indicating that the baseline diabetic phenotype remained the dominant contributor to the overall morphology-based profile (Fig. 2d).

At the feature level, unsupervised clustering of the top 50 regulated morphological parameters further supported these observations. Healthy control cells displayed a coordinated pattern of nuclear, RNA-associated, and membrane-related features that differed from that observed in T2D-derived cells. BPS treatment modified this profile in healthy cells, with several features related to RNA distribution, Golgi membrane intensity, and perinuclear organization showing changes partially overlapping with features that distinguished T2D-derived cells. In contrast, T2D-derived cells showed more limited overall responsiveness to BPS, with smaller changes relative to untreated T2D-derived controls. Nevertheless, among the features most affected by BPS in T2D-derived cells, several were related to RER morphology, suggesting a more restricted compartment-specific response under diabetic conditions (Fig. 2e).

Taken together, these results indicate that metabolic status is the major determinant of the endothelial morphology-based phenotype. BPS exposure induces a pronounced multidimensional phenotypic shift in healthy endothelial cells, whereas T2D-derived cells retain a more stable disease-associated profile, with evidence of selective RER-associated responsiveness to BPS.

### Golgi membrane features are primarily shaped by metabolic status and are not significantly modified by BPS treatment

**Fig. 3:**
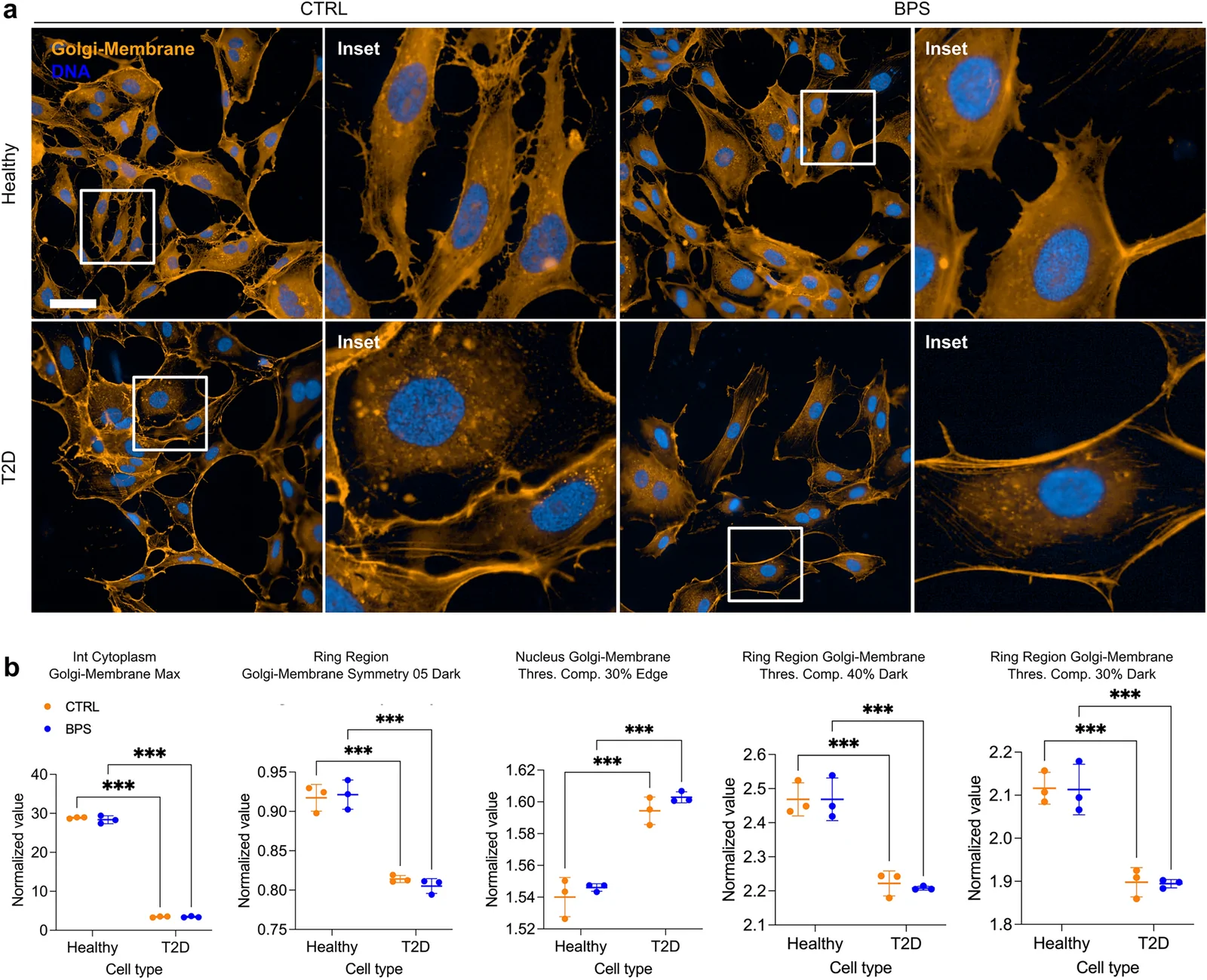
Membrane-associated (Golgi membrane) features in healthy and T2D cells under CTRL and BPS conditions. **a** Representative fluorescence images of the Golgi membrane channel across experimental groups: healthy CTRL, healthy BPS, T2D CTRL, and T2D BPS cells. Scale bar, 50 µm; 40 × objective. **b** Quantitative analysis of selected Golgi membrane morphological features extracted from high-content imaging. Data are presented as mean ± SD from three independent biological replicates. Statistical analysis was performed using two-way ANOVA with cell type and treatment as factors. ***p < 0.001

Quantitative analysis of membrane-associated features revealed a robust effect of cell type, with multiple descriptors of the Golgi membrane compartment significantly differing between healthy cells and T2D cells (Fig. 3a).

Features capturing signal intensity, spatial distribution, and symmetry within membrane-related regions—including Int Cytoplasm Golgi-Membrane Max, Ring Region Golgi-Membrane Symmetry 05 Dark, Ring Region Golgi-Membrane Threshold Compartment (30–40%), and Nucleus Golgi-Membrane Threshold Compartment (30% Edge)—were significantly influenced by cell type (*p* < 0.001). In contrast, neither the main effect of treatment (BPS) nor the cell type × treatment interaction reached statistical significance for these variables (Fig. 3b).

These data indicate that the selected Golgi membrane-associated features are primarily determined by the underlying metabolic condition, with stable differences between healthy and T2D-derived endothelial cells. Conversely, BPS exposure did not induce measurable changes in this specific subset of features under the experimental conditions tested. Thus, although Golgi membrane descriptors contributed to the multivariate discrimination of endothelial phenotypes, their variation was mainly attributable to metabolic status rather than to BPS treatment.

### RER-associated features reveal metabolic status-dependent and BPS-responsive remodeling

**Fig. 4:**
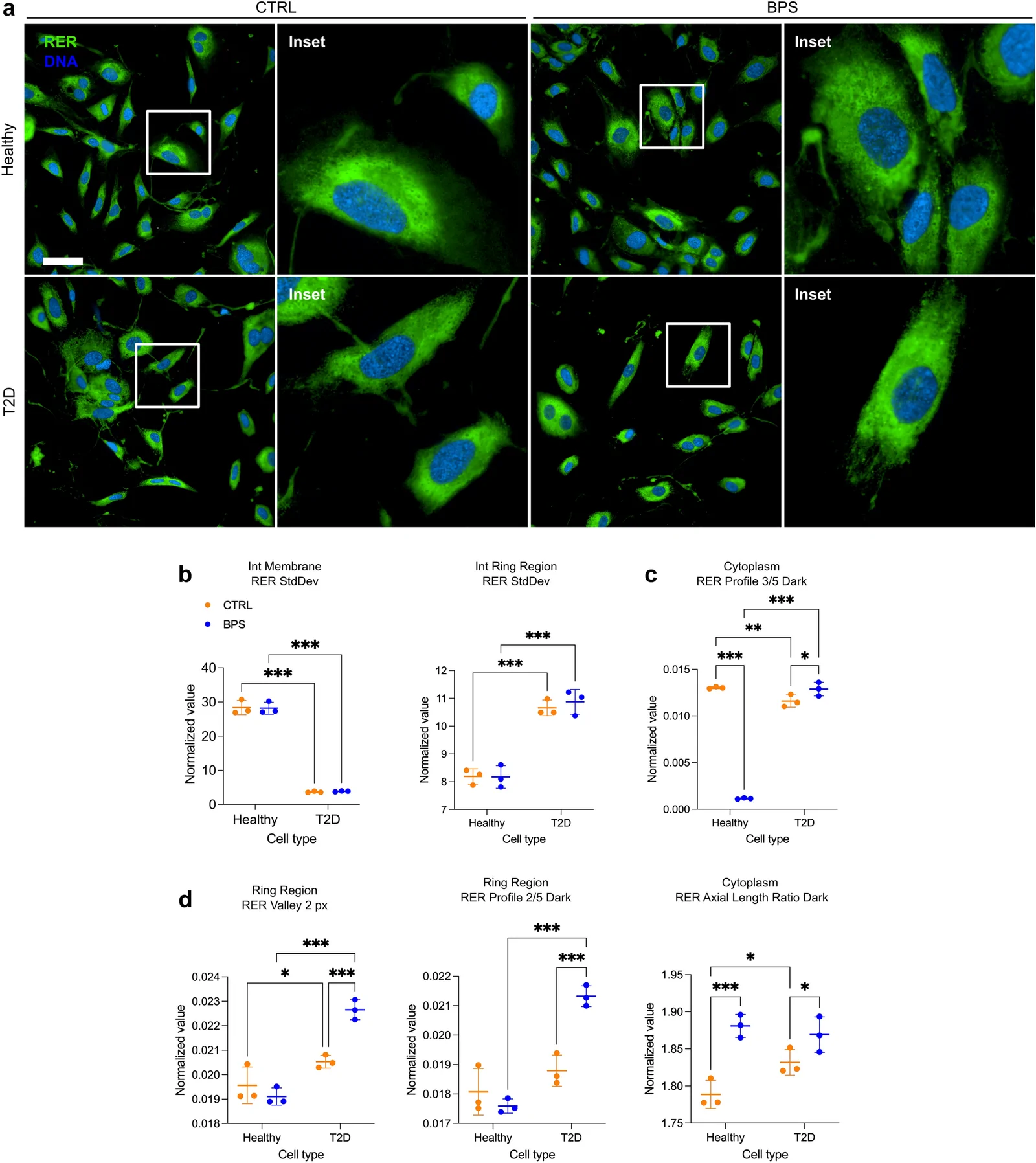
Rough endoplasmic reticulum (RER)-associated features in healthy and T2D cells under CTRL and BPS conditions. **a** Representative fluorescence images of the RER channel across experimental groups. Scale bar 50 µm, 40× objective. **b** RER-associated features significantly affected by cell type, showing differences between healthy and T2D-derived cells independently of treatment. **c** RER-associated feature characterized by a significant cell type × treatment interaction, indicating a differential response to BPS according to metabolic status. **d** RER-associated features showing BPS-related modulation mainly in T2D-derived cells, with significant differences between CTRL and BPS conditions under diabetic conditions and minimal changes in healthy cells. Data are presented as mean ± SD from three independent biological replicates. Statistical analysis was performed using two-way ANOVA with cell type and treatment as factors. *p < 0.05, **p < 0.01, ***p < 0.001

To further investigate compartment-specific alterations associated with BPS exposure, we analyzed morphological features related to the RER, a key compartment involved in protein synthesis and cellular stress responses. Representative images showed differences in RER signal organization across experimental conditions, including changes in intracellular distribution and texture between healthy and T2D-derived endothelial cells, together with more subtle BPS treatment-associated variations (Fig. 4a). Quantitative analysis identified distinct patterns according to the contribution of cell type, BPS treatment, and their interaction.

Several RER-associated features were primarily influenced by cell type, showing significant differences between healthy and T2D-derived endothelial cells independently of BPS treatment (Fig. 4b). These features, including descriptors of RER intensity variability and spatial distribution, such as Membrane RER StdDev and Ring Region RER StdDev, showed a consistent separation between healthy and T2D-derived cells, with no significant main effect of BPS.

In contrast, Cytoplasm RER Axial Length Ratio Dark showed a significant cell type × treatment interaction, indicating that the effect of BPS differed according to metabolic status (Fig. 4c). Specifically, BPS decreased this feature in healthy cells, whereas it increased it in T2D-derived cells, resulting in divergent treatment-associated trends between the two cell types. This pattern suggests a context-dependent modulation of RER organization by BPS.

A further subset of RER-associated features showed BPS-related modulation that was mainly evident in T2D-derived cells (Fig. 4d). These variables, including RER profile and valley texture descriptors in perinuclear/ring and cytoplasmic regions, differed between CTRL and BPS conditions in T2D-derived cells while remaining largely unchanged in healthy cells. This finding indicates that selected RER-associated parameters retain sensitivity to BPS exposure under diabetic conditions.

Overall, these results indicate that RER morphology is influenced by metabolic status but also remains responsive to BPS in a context-dependent manner. In particular, RER-associated descriptors revealed both interaction-dependent effects and T2D-specific treatment-related changes, suggesting that the RER may represent a selectively responsive compartment in diabetic endothelial cells.

### RNA-associated features reveal dominant cell type effects with additional interaction-dependent modulation

To further characterize disease- and treatment-associated phenotypic changes, we analyzed morphology-related features associated with RNA-rich regions, which provide information on intracellular RNA organization and RNA-associated processes linked to gene expression and protein synthesis.

**Fig. 5:**
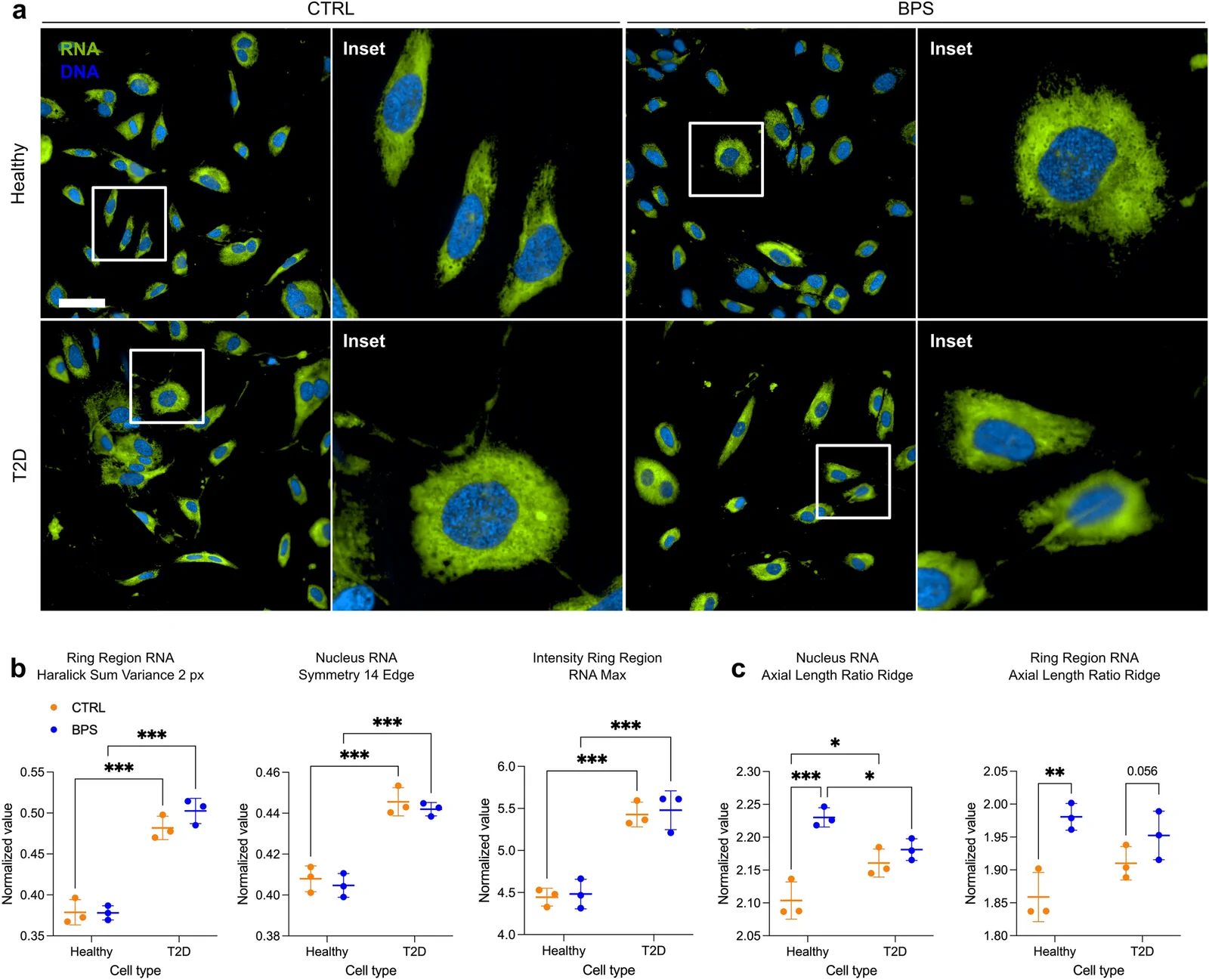
RNA-associated features in healthy and T2D under CTRL and BPS conditions. **a** Representative fluorescence images of the RNA channel showing intracellular signal distribution across experimental groups: healthy CTRL, healthy BPS, T2D CTRL, and T2D BPS cells. Scale bar, 50 µm; 40 × objective. **b** Features significantly affected by cell type, showing differences between healthy and T2D-derived cells independently of treatment. **c** Features characterized by a significant cell type × treatment interaction, indicating a differential response to BPS according to metabolic status. Data are presented as mean ± SD from three independent biological replicates. Data are presented as mean ± SD from three independent biological replicates. Statistical analysis was performed using two-way ANOVA with cell type and treatment as factors. *p < 0.05, **p < 0.01, ***p < 0.001

Representative images showed differences in RNA signal distribution across experimental conditions, particularly in perinuclear and cytoplasmic regions, between healthy and T2D-derived endothelial cells (Fig. 5a). Quantitative analysis identified a predominant contribution of cell type, with several RNA-associated features significantly differing between healthy and T2D-derived cells independently of BPS treatment (Fig. 5b). These included descriptors of RNA signal intensity, symmetry, and texture, such as Intensity Ring Region RNA Max, RNA symmetry features, and Haralick texture descriptors. For these variables, CTRL and BPS conditions largely overlapped within each cell type, indicating that the RNA-associated morphology-based profile was mainly determined by metabolic status.

In addition, a subset of RNA-associated features showed a significant cell type × treatment interaction (Fig. 5c), indicating that BPS-related changes differed according to metabolic condition. For these parameters, BPS induced modest but divergent changes in healthy and T2D-derived cells, as reflected by non-parallel trends between the CTRL and BPS groups. Although these effects were smaller than the differences associated with cell type, they suggest a context-dependent modulation of RNA-associated intracellular organization by BPS.

Overall, RNA-associated features reinforce the dominant contribution of metabolic status to endothelial morphology, while also revealing subtle interaction-dependent effects of BPS detectable by high-content imaging.

### Mitochondrial features show BPS-dependent modulation mainly in healthy arterial endothelial cells

**Fig. 6:**
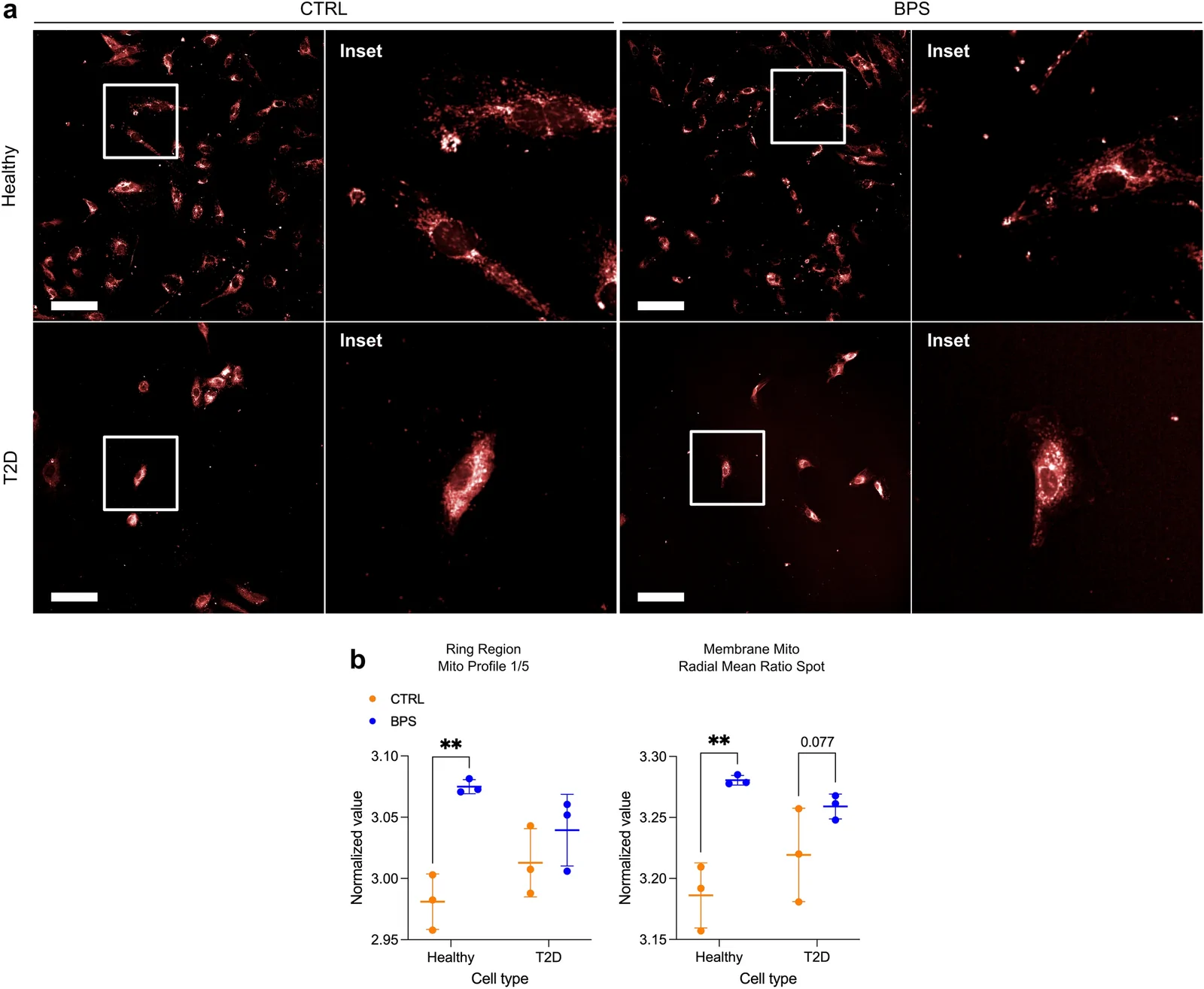
Mitochondria-associated features in healthy and T2D under CTRL and BPS conditions. **a** Representative fluorescence images of the mitochondrial channel showing intracellular organization across experimental groups (healthy CTRL, healthy BPS, T2D CTRL, T2D BPS cells). Insets show magnified regions of interest. Scale bar 100 µm, 20× objective. **b** Quantitative analysis of selected mitochondrial morphological features extracted from high-content imaging. Data are presented as mean ± SD from three independent biological replicates. Statistical analysis was performed using two-way ANOVA with cell type and treatment as factors, followed by post hoc comparisons when appropriate. Ring Region Mito Profile 1/5 showed a significant treatment effect and a significant cell type × treatment interaction, with a significant increase in BPS-treated healthy, compared with CTRL. **p < 0.01

Representative mitochondrial images showed differences in signal organization across experimental groups (Fig. 6a). In healthy CTRL cells, the mitochondrial signal appeared broadly distributed throughout the cytoplasm, with a recognizable perinuclear and elongated pattern. After BPS exposure, healthy cells showed a more regionally accentuated mitochondrial signal. In T2D-derived cells, the mitochondrial signal appeared more compact and less widely distributed, with comparatively smaller changes after BPS treatment.

Quantitative analysis identified two image-derived mitochondrial descriptors significantly influenced by BPS exposure (Fig. 6b). Ring Region Mito Profile 1/5, reflecting mitochondrial signal distribution within the perinuclear/ring region, showed a significant main effect of treatment and a significant cell type × treatment interaction. Post hoc analysis revealed a significant increase in BPS-treated healthy cells compared with healthy CTRL cells, whereas no significant difference was observed between CTRL and BPS conditions in T2D-derived cells. Membrane Mito Radial Mean Ratio Spot, reflecting membrane-associated radial distribution of the mitochondrial signal, showed a significant main effect of treatment, while the interaction did not reach statistical significance. Post hoc comparisons indicated a significant increase in healthy cells following BPS exposure, whereas T2D-derived cells showed only a trend toward significance.

Overall, these data indicate that BPS modifies selected mitochondrial spatial distribution features mainly in healthy endothelial cells, while mitochondrial-associated parameters in T2D-derived cells show a weaker treatment-related response.

### Nuclear DNA features reveal BPS-dependent changes mainly in healthy endothelial cells

**Fig. 7:**
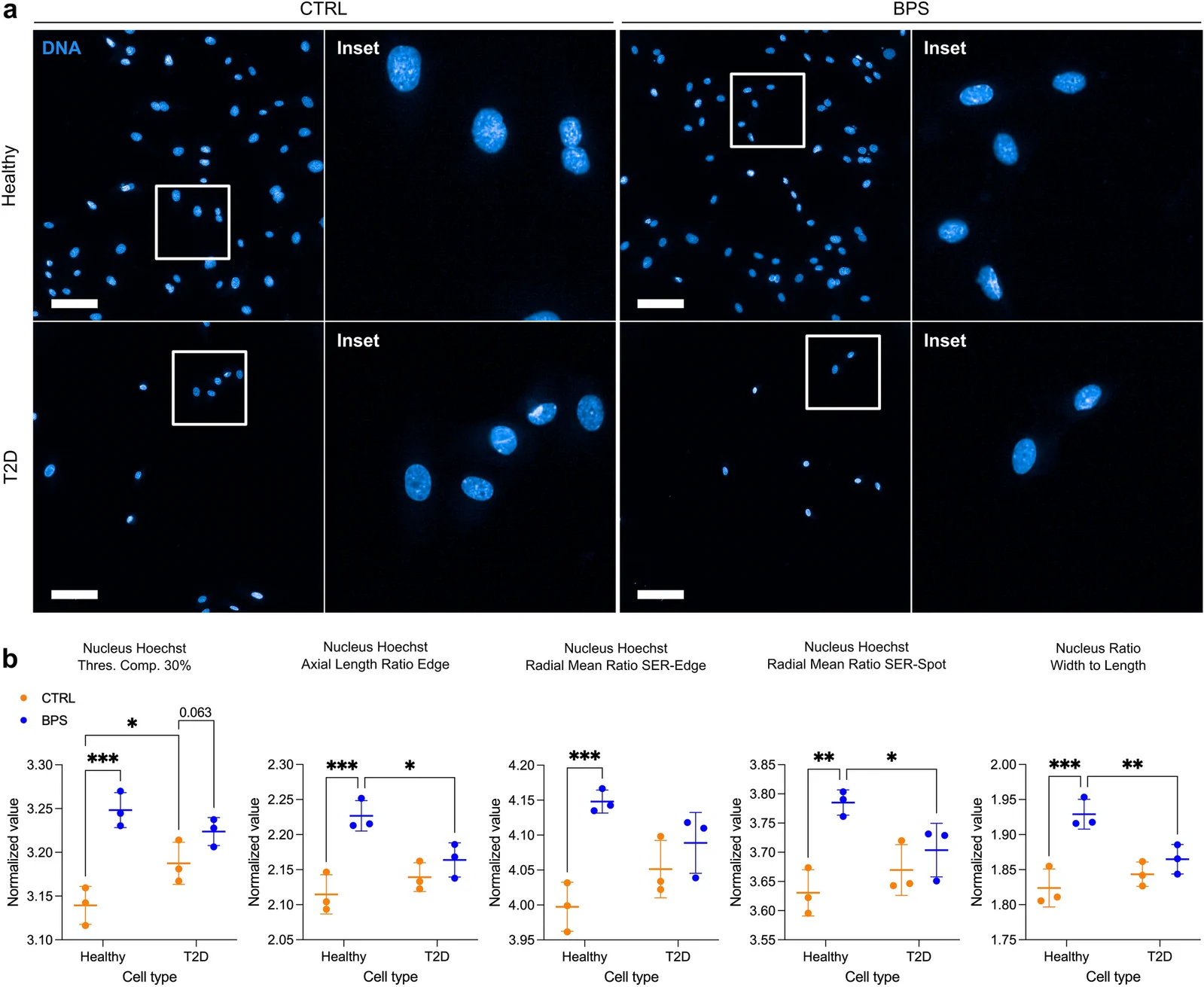
Nuclear DNA-associated features in healthy and T2D under CTRL and BPS conditions. **a** Representative fluorescence images of the DNA/Hoechst channel showing nuclear staining across experimental groups (healthy CTRL, healthy BPS, T2D CTRL, T2D BPS cells). Insets show magnified regions of interest. **b** Quantitative analysis of selected nuclear morphological and Hoechst signal distribution features extracted from high-content imaging. Data are presented as mean ± SD from three independent biological replicates. Statistical analysis was performed using two-way ANOVA with cell type and treatment as factors, followed by post hoc comparisons when appropriate. BPS significantly increased multiple nuclear parameters in healthy, including threshold compartment, axial length ratio, radial mean ratio, and width-to-length ratio features, while T2D showed limited treatment-related changes. *p < 0.05, **p < 0.01, ***p < 0.001

Hoechst-derived images and quantitative descriptors revealed BPS-associated changes in nuclear morphology and signal distribution, mainly in healthy endothelial cells (Fig. 7a, b). In healthy cells, BPS increased multiple parameters related to nuclear signal distribution and nuclear shape, including threshold compartment, axial length ratio, radial mean ratio, and width-to-length ratio features. In contrast, T2D-derived cells showed a more limited response, with only a trend observed for one nuclear parameter. Significant differences between healthy and T2D-derived cells under BPS treatment further indicated that the nuclear response to BPS was strongly influenced by baseline metabolic status. Overall, these results indicate that BPS affects nuclear morphology and Hoechst signal distribution features mainly in healthy endothelial cells.

## Discussion

In this study, we used high-content imaging and Cell Painting to investigate whether exposure to 0.1 µM BPS, selected to model exposure-relevant low-dose conditions, modifies morphology-based phenotypic features of primary human arterial endothelial cells according to baseline metabolic status. The main findings are that metabolic status was the strongest determinant of the endothelial morphology-based profile; BPS induced a marked multidimensional phenotypic shift in healthy endothelial cells; T2D-derived cells retained a dominant disease-associated profile with a less evident whole-cell response to BPS; selected RER-associated features remained responsive to BPS in T2D-derived cells. Together, these findings indicate that BPS perturbs endothelial morphology under low-dose exposure-relevant conditions, while the magnitude and distribution of this response are strongly shaped by the pre-existing metabolic condition.

The clear separation between healthy and T2D-derived endothelial cells indicates that the diabetic condition is associated with a broader reorganization of the endothelial morphology-based phenotype. This observation is consistent with previous evidence showing that endothelial cells exposed to diabetic conditions retain persistent structural and functional alterations driven by chronic metabolic stress (Zhang et al. 2012; Yao et al. 2022; Fang et al. 2024).

In this context, the multivariate separation observed in our study may reflect disease-associated morphological imprinting, in which organelle distribution, membrane organization-related features, nuclear descriptors, and stress-associated compartments collectively contribute to the diabetic endothelial phenotype (Yao et al. 2022; Gorashi et al. 2023).

This baseline remodeling is biologically relevant because it may influence how endothelial cells respond to additional environmental challenges.

A central result of this study is that BPS exposure induced a pronounced morphology-based phenotypic shift in healthy endothelial cells. This finding supports the view that BPS is not an inert BPA substitute, but a biologically active pollutant capable of altering endothelial cell organization under low-dose exposure conditions (Rochester and Bolden 2015; Wu et al. 2018; Hu et al. 2024). However, the present data do not establish whether these changes result in functional endothelial impairment, thus paving the way for future functional assessments.

In healthy cells, BPS-sensitive features involved nuclear descriptors, mitochondrial spatial distribution parameters, RNA-associated organization, and membrane/perinuclear compartments. Although these morphological changes do not directly demonstrate functional impairment, they indicate that BPS can perturb multiple subcellular domains involved in endothelial homeostasis (Zhang et al. 2024).

This is consistent with previous studies showing that BPS and related plastic-associated pollutants can induce mitochondrial, transcriptional, and phenotypic alterations in human cell-based models, even in the absence of overt cytotoxicity (Gaggi et al. 2023; Di Credico et al. 2023a).

The observation that BPS-treated healthy cells partially shifted along morphological axes that also distinguished T2D-derived cells suggests that BPS exposure can move otherwise healthy endothelial cells toward a profile sharing selected features with the diabetic endothelial phenotype. This convergence supports the possibility that environmental pollutant exposure and metabolic disease affect common subcellular domains, including mitochondrial organization, nuclear architecture, RNA-associated intracellular organization, and membrane-related remodeling (Easson et al. 2022; Fang et al. 2024).

From a vascular-risk perspective, these early morphology-based alterations reveal endothelial sensitivity to BPS under exposure-relevant conditions and suggest a potential route through which environmental pollutants could contribute to endothelial vulnerability.

In T2D-derived endothelial cells, the overall BPS-associated shift was less evident because the baseline morphology-based profile was already dominated by disease-associated alterations. Thus, while BPS clearly shifted healthy endothelial cells away from their basal phenotype, its additional impact on T2D-derived cells was less apparent at the global multivariate level, where a pathology-based morphology profile was already established. Under these conditions, BPS responsiveness emerged more clearly at the level of selected descriptors than as a broad whole-cell phenotypic shift (Zhang et al. 2012; Yao et al. 2022; Fang et al. 2024).

This interpretation is supported by the analysis of RER-associated features. Several RER descriptors were influenced by metabolic status, whereas others showed significant interaction-dependent or T2D-specific BPS-related changes. Thus, although the global morphology-based profile of T2D-derived cells remained largely dominated by the diabetic condition, RER-associated parameters revealed sensitivity to BPS exposure. This finding is biologically relevant because the RER is closely linked to protein synthesis, proteostasis, and cellular stress responses, processes frequently dysregulated in metabolic disease (Özcan et al. 2004; Lenna et al. 2014; Hetz et al. 2020).

In diabetic endothelial cells, hyperglycemia, insulin resistance, oxidative stress, and inflammatory stimuli can activate ER stress pathways and contribute to endothelial dysfunction (Magenta et al. 2014). The modulation of RER-associated descriptors by BPS is therefore consistent with an additional perturbation of stress-related subcellular organization in an already compromised endothelial phenotype (Hu et al. 2024).

The Golgi membrane and RNA-associated results further support the dominant role of metabolic status. Golgi membrane-associated features contributed to the multivariate discrimination of endothelial phenotypes, but their variation was mainly attributable to cell type rather than to BPS treatment. Similarly, RNA-associated features were largely determined by cell type, with additional interaction-dependent modulation by BPS. These findings indicate that the diabetic condition establishes a stable intracellular organization involving membrane-associated and RNA-rich compartments, while BPS-related changes emerge more selectively depending on cellular context (Bexiga and Simpson 2013; Cancino et al. 2013).

Mitochondrial and nuclear descriptors provided complementary evidence of BPS sensitivity, especially in healthy endothelial cells. BPS significantly altered selected mitochondrial spatial distribution features and several Hoechst-derived nuclear parameters in healthy cells, whereas T2D-derived cells showed a weaker or more limited response. These findings are consistent with the known involvement of mitochondrial and nuclear alterations in both endothelial dysfunction and EDC-related toxicity (Gorashi et al. 2023) and fit with previous evidence from human stem cell-based models showing that BPS and related plastic-associated pollutants, tested under low-dose exposure-relevant conditions, can affect mitochondrial homeostasis and deregulate genes involved in epigenetic regulation (Gaggi et al. 2023, 2025; Di Credico et al. 2023a).

In the present study, mitochondrial and Hoechst-derived descriptors expand this evidence by identifying morphology-based signatures of BPS-induced cellular perturbation in human arterial endothelial cells. Further studies in endothelial models will help define how these phenotypic changes relate to mitochondrial activity, chromatin-related regulation, and downstream endothelial responses.

Taken together, these findings support the hypothesis that BPS may act as an additional environmental stressor for the endothelium. In healthy endothelial cells, BPS induced early morphology-based alterations across multiple compartments under low-dose exposure-relevant conditions. In T2D-derived cells, where a disease-associated phenotype was already established, BPS effects were less evident as a global shift but remained detectable in stress-sensitive compartments, particularly the RER. This pattern suggests that environmental pollutants may contribute to endothelial vulnerability by perturbing otherwise healthy cells and further compromising cells already compromised by metabolic disease.

A strength of this study is the use of Cell Painting combined with multivariate and inferential statistics. This strategy enabled the detection of coordinated morphology-based signatures across multiple compartments and helped distinguish the dominant contribution of metabolic status from the more selective, context-dependent effects of BPS. Multivariate analysis captured overall phenotypic relationships among experimental groups, whereas feature-level statistics identified the compartments and descriptors mainly associated with cell type, treatment, or their interaction (Mattiazzi Usaj et al. 2016; Bray et al. 2016; Vulliard et al. 2022).

Several limitations should be acknowledged. First, this is an in vitro study and cannot fully reproduce the complexity of the in vivo vascular environment, including hemodynamic forces, immune cell interactions, circulating metabolites, and chronic systemic inflammation. Second, Cell Painting provides high-dimensional morphological information but does not directly identify the molecular mechanisms underlying the observed changes. Therefore, the links between BPS-induced morphological remodeling, endothelial dysfunction, ER stress, mitochondrial activity, and inflammatory signaling remain to be experimentally validated. Accordingly, the next step could include a targeted mechanistic and functional validation.

Third, the study was performed at a single BPS concentration and exposure time; dose-response and time-course experiments will be required to determine whether the observed effects are transient, progressive, or adaptive. Finally, future studies should consider donor sex and sex-specific hormone signaling, since EDC effects and cardiovascular susceptibility may differ according to biological sex (Assenza et al. 2025; Gaggi et al. 2025).

In conclusion, our findings show that BPS exposure induces a marked morphology-based phenotypic shift in healthy arterial endothelial cells, supporting its biological activity as an environmental endocrine-disrupting pollutant. In T2D-derived endothelial cells, the dominant disease-associated phenotype reduced the global detectability of BPS-induced remodeling, while selected compartments, particularly the RER, remained responsive. These data highlight the importance of considering baseline metabolic status when evaluating endothelial responses to environmental pollutants and support further functional studies to determine whether BPS contributes to endothelial vulnerability and cardiovascular risk in metabolically compromised individuals.

## Supplementary Information

Below is the link to the electronic supplementary material.

Supplementary file1 (TIFF 39135 KB)

## Data Availability

Data are available on reasonable request from the authors.
